# Genome-Wide Identification and Characterization of Carboxypeptidase Genes in Silkworm (*Bombyx mori*)

**DOI:** 10.3390/ijms17081203

**Published:** 2016-07-28

**Authors:** Junhong Ye, Yi Li, Hua-Wei Liu, Jifu Li, Zhaoming Dong, Qingyou Xia, Ping Zhao

**Affiliations:** State Key Laboratory of Silkworm Genome Biology, Southwest University, Chongqing 400716, China; yejunhong0129@outlook.com (J.Y.); yili89716@gmail.com (Y.L.); lhw888718@163.com (H.-W.L.); lijifu0103@163.com (J.L.); dong-zhaoming@163.com (Z.D.); xiaqy@swu.edu.cn (Q.X.)

**Keywords:** carboxypeptidase family, evolutionary analysis, expression pattern, starvation, *Bombyx mori*

## Abstract

The silkworm (*Bombyx mori*) is an economically-important insect that can secrete silk. Carboxypeptidases have been found in various metazoan species and play important roles in physiological and biochemical reactions. Here, we analyzed the silkworm genome database and characterized 48 carboxypeptidases, including 34 metal carboxypeptidases (*BmMCP1*–*BmMCP34*) and 14 serine carboxypeptidases (*BmSCP1*–*BmSCP14*), to better understand their diverse functions. Compared to other insects, our results indicated that carboxypeptidases from silkworm have more family members. These silkworm carboxypeptidases could be divided into four families: Peptidase_M2 carboxypeptidases, Peptidase_M14 carboxypeptidases, Peptidase_S10 carboxypeptidases and Peptidase_S28 carboxypeptidases. Microarray analysis showed that the carboxypeptidases had distinct expression patterns, whereas quantitative real-time PCR demonstrated that the expression level of 13 carboxypeptidases significantly decreased after starvation and restored after re-feeding. Overall, our study provides new insights into the functional and evolutionary features of silkworm carboxypeptidases.

## 1. Introduction

In insects, food proteins are preliminary digested by midgut endopeptidases and then by exopeptidases into single free amino acids that are further absorbed by intestinal cells [1]. Endopeptidases, such as trypsin, chymotrypsin, elastase, thermolysin, pepsin, glutamyl endopeptidase, cathepsin B, cathepsin L and neprilysin, are proteolytic peptidases that break the peptide bonds of nonterminal amino acids. On the other hand, exopeptidases, such as aminopeptidases and carboxypeptidases, are applied to the N-terminal and C-terminal peptide bonds, respectively, of the protein to release single free amino acids [2]. Carboxypeptidases are classified into two sub-categories, according to their catalytic mechanism: serine carboxypeptidases with an active serine residue in the active site and metal carboxypeptidases with a metal ion in the active site [3,4]. In general, carboxypeptidases perform important physiological functions, such as food digestion, blood clotting, growth factor production and regulation of biological processes in tissues and organs [5,6,7].

Carboxypeptidases have been identified in many insects, including *Simulium vittatum* [8], *Glossina morsitans* [9], *Helicoverpa armigera* [10], *Mamestra configurata* [11], *Anopheles gambiae* [12], *Aedes aegypti* [13], *Trichoplusia ni* [1,14] and *Triatoma brasiliensis* [15]. In 1976, Ward first reported carboxypeptidase activity in the midgut of *Tineola bisselliella* [16]. In 1997, Broadway identified five types of carboxypeptidases in the midgut of *Trichoplusia ni* [14] and reported that carboxypeptidase A is eight-fold more active than carboxypeptidase B [1]. Eighteen carboxypeptidase genes were identified in *Aedes aegypti*, whereas after blood feeding, the expression level of 11 carboxypeptidase genes increased 40-fold in the midgut [7]. In blood-sucking insect populations, the expression of digestive carboxypeptidase genes is promoted by blood meal [8,12].

The silkworm is commonly known as an economically-important insect that secretes silk protein threads to build a cocoon. The study of protein digestion and nutrient absorption in silkworm may reveal the underlying mechanism of silk protein synthesis. Most of the silkworm endopeptidases are serine proteases. In 2010, 51 serine proteases and 92 serine proteases homologs were identified in the silkworm [17]. However, information on carboxypeptidases is still limited. The carboxypeptidase gene MF-CPA from silkworm molting fluid has been previously cloned, characterized [18] and identified in the embryos at the end of the organogenesis [19]. Since silkworm genome sequencing is completed, it is possible to identify the carboxypeptidases family members in the whole genome of silkworm, *B. mori* [20,21,22]. In the present work, we identified the silkworm carboxypeptidases family and analyzed its genomic organization, expression and molecular evolution in order to reveal additional unknown carboxypeptidases. Moreover, we investigated the characteristics of the silkworm carboxypeptidase family, including phylogeny relationships, as well as spatial and temporal expression profiles. Our results provided preliminary evidence to support the functional roles of carboxypeptidases in food digestion.

## 2. Results

### 2.1. Identification and Characterization of the Carboxypeptidase Family

By searching the silkworm genome database (SilkDB), 48 members of the carboxypeptidase family were identified; these included, 34 metal carboxypeptidases (nine metal carboxypeptidases containing the Peptidase_M2 domain and twenty five metal carboxypeptidases containing the Peptidase_M14 domain) and 14 serine carboxypeptidases (five serine carboxypeptidases containing the Peptidase_S10 domain and nine serine carboxypeptidases containing the Peptidase_ S28 domain; Table 1).

### 2.2. Phylogenetic Analysis

**Figure 1:**
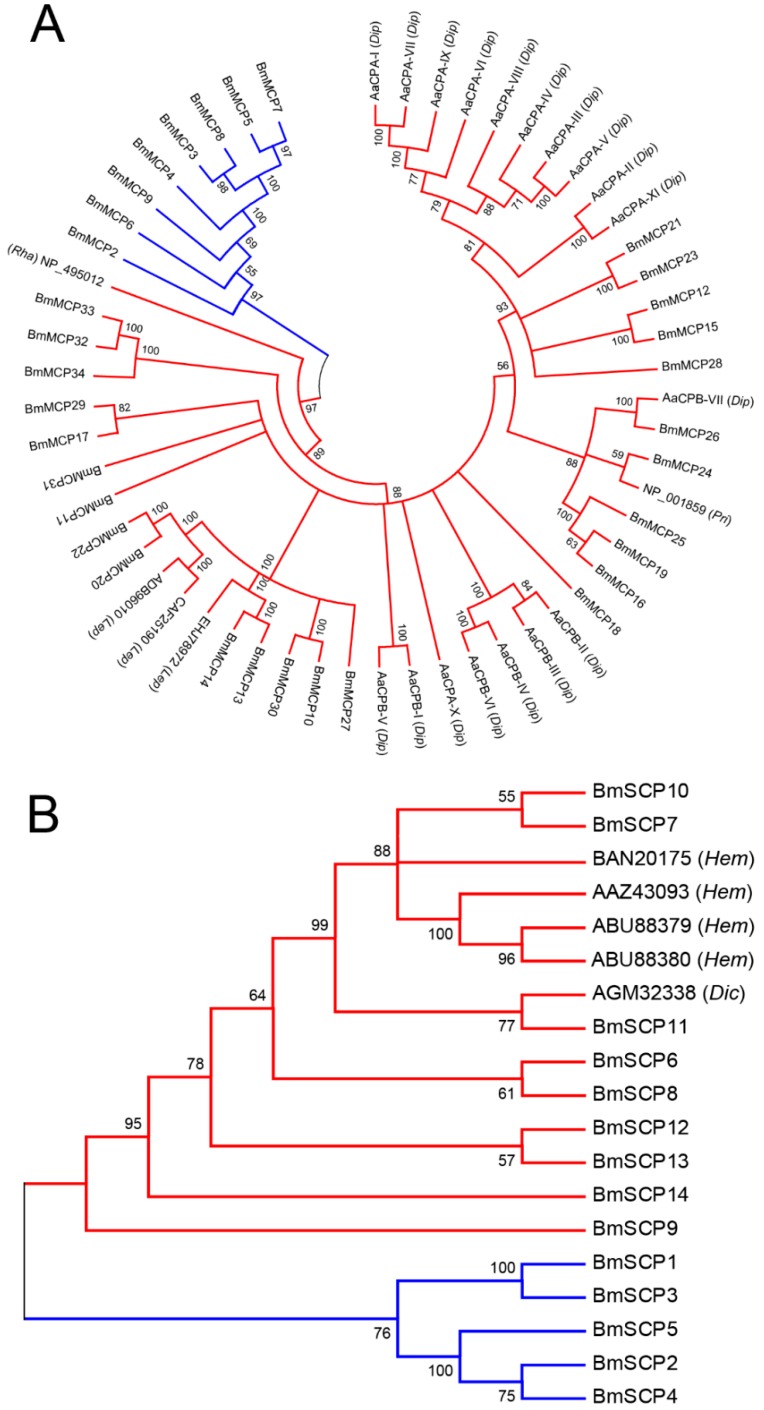
Phylogenetic analysis of carboxypeptidase amino acids. (**A**) Neighbor-joining tree of the metal carboxypeptidase family. Red lines represent metal carboxypeptidases with the Peptidase_M14 domain, and blue lines represent metal carboxypeptidases with the Peptidase_M2 domain; (**B**) Neighbor-joining tree for the serine carboxypeptidase family. Red lines represent serine carboxypeptidases with the Peptidase_S10 domain, and blue lines represent serine carboxypeptidases with the Peptidase_S28 domain. The number at each branch represents the percentage of 1000 bootstrap iterations. Values below 50% were omitted. Abbreviations: *Rha*, *Rhabditida*; *Pri*, *Primates*; *Lep*, *Lepidoptera*; *Dip*, *Diptera*; *Hem*, *Hemiptera*; *Dic*, *Dictyoptera*.

Carboxypeptidases are usually classified into two sub-categories based on their active sites: serine carboxypeptidases and metal carboxypeptidases [3]. To analyze the relationship between silkworm metal carboxypeptidases and those of other species, a phylogenetic tree was constructed with MEGA 6.0, using metal carboxypeptidase amino acid sequences from silkworm and *Caenorhabditis elegans* (NP_495012), *Aedes aegypti* (AAT36725.1, AAT36726.1, AAT36727.1, AAT36728.1, AAT36729.1, AAT36730.1, AAT36731.1, EAT39608, EAT37218, EAT46817, EAT44906, AAT36732.1, AAT36733.1, AAT36734.1, ABO21075.1, ABO21076.1, ABO21077.1 and EAT36173), *Danaus plexippus* (EHJ78972), *Spodoptera frugiperda* (ADB96010), *Helicoverpa armigera* (CAF25190) and *Homo sapiens* (NP_001859). Metal carboxypeptidases with different domains (Peptidase_M2 and Peptidase_M14) were clustered into different groups based on their evolutionary relationship (Figure 1A). Moreover, phylogenetic analyses showed that metal carboxypeptidases with the Peptidase_M14 domain in silkworm were clustered together with those of other species. We also constructed a phylogenetic tree using serine carboxypeptidase amino acid sequences from silkworm and *Triatoma brasiliensis* (ABU88379 and ABU88380), *Triatoma infestans* (AAZ43093), *Coptotermes formosanus* (AGM32338) and *Riptortus pedestris* (BAN20175). The results showed that serine carboxypeptidase duplication and divergence led to the separation of Peptidase_S10 carboxypeptidases and Peptidase_S28 carboxypeptidases (Figure 1B) and that silkworm Peptidase_S10 carboxypeptidases have a very close genetic relationship with those of the other insect species.

### 2.3. Expression Profiles

**Figure 2:**
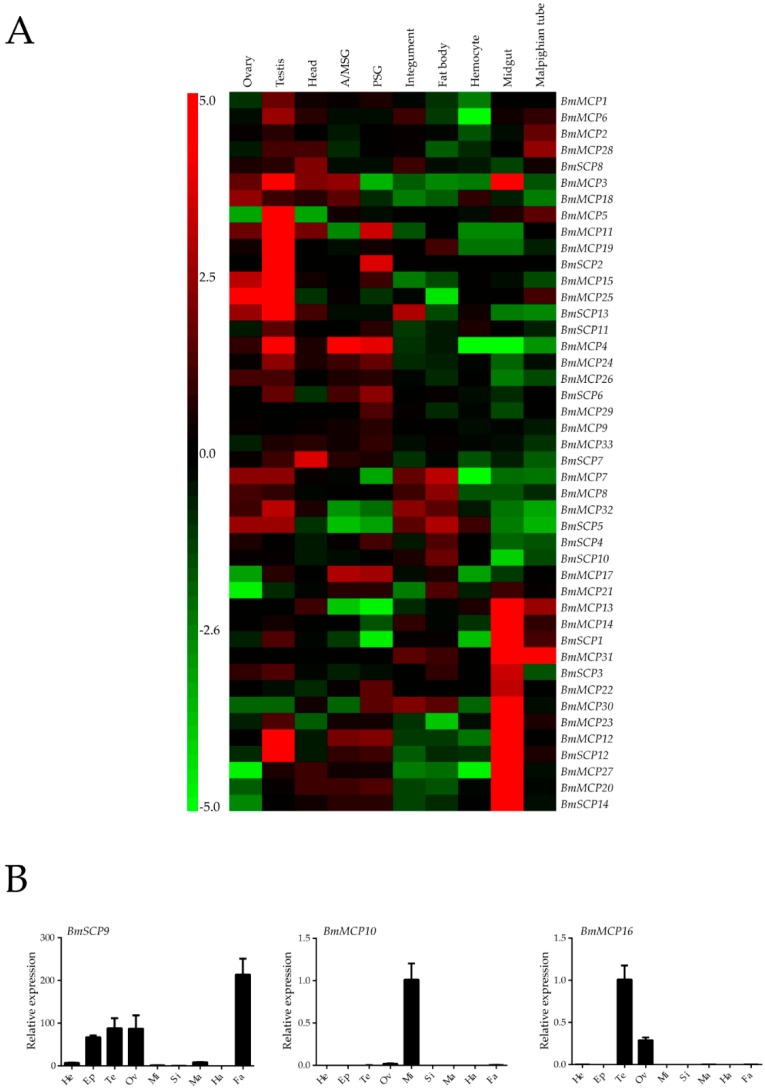
(**A**) Microarray analysis of carboxypeptidase expression in different silkworm tissues. Each column represents an organ or tissue, including testis, ovary, head, integument, fat body, midgut, hemocytes, Malpighian tubules, anterior/middle silk gland and posterior silk gland. Red represents high expression, and green represents low expression; (**B**) Expression patterns of the silkworm carboxypeptidases *BmSCP9*, *BmMCP10* and *BmMCP16*. Abbreviations: He, head; Ep, epidermis; Te, testis; Ov, ovary; Mi, midgut; Si, silk gland; Ma, Malpighian tubules; Ha, hemocytes; Fa, fat body.

Microarray data from SilkDB were used to analyze the expression profiles of carboxypeptidases in different tissues. As shown in Figure 2A, 44 carboxypeptidases had corresponding oligonucleotide probes, and a heat map was created based on signal intensity values. The majority of Peptidase_M14 carboxypeptidases was highly expressed in the midgut, whereas other high expression levels in the testis. The rest of carboxypeptidases were expressed in various tissues (Figure 2A).

The expression profiles of four carboxypeptidase genes (*BmMCP10*, *BmMCP16*, *BmMCP34* and *BmSCP9*) that lack specific oligonucleotide probes in the database were replenished by quantitative real-time PCR (qRT-PCR). *BmSCP9* was mainly expressed in the fat body epidermis and sex gland. *BmMCP10* was highly expressed in the midgut. *BmMCP16* was expressed in the sex gland. However, the gene *BmMCP34* was not expressed in all tissues at Day 3 of the fifth instar larval stage (Figure 2B).

**Figure 3:**
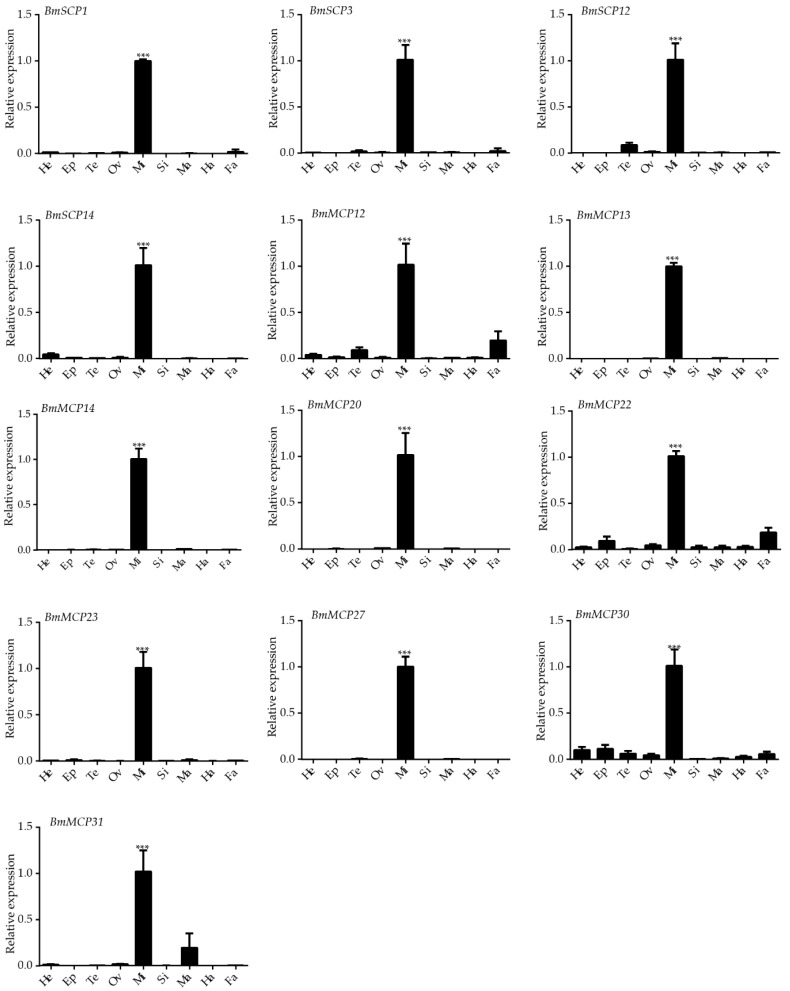
Expression patterns of midgut-specific carboxypeptidases at Day 3 of the fifth instar. Abbreviations: He, head; Ep, epidermis; Te, testis; Ov, ovary; Mi, midgut; Si, silk gland; Ma, Malpighian tubules; Ha, hemocytes; Fa, fat body. *** *p* < 0.001.

The expression profile of the midgut-specific carboxypeptidases was further examined by qRT-PCR in nine different tissues, including the head, epidermis, testis, ovary, midgut, silk gland, Malpighian tubules, hemocytes and fat body at Day 3 of the fifth instar larval stage (Figure 3). The results were consistent with those of the heat map.

Moreover, we created two temporal expression pattern heat maps, one for each gender (Figure S1). Midgut-specific carboxypeptidases were expressed throughout the larval stage, whereas others were only expressed in the pupal stage.

### 2.4. Carboxypeptidase Expression Profile after Starvation

**Figure 4:**
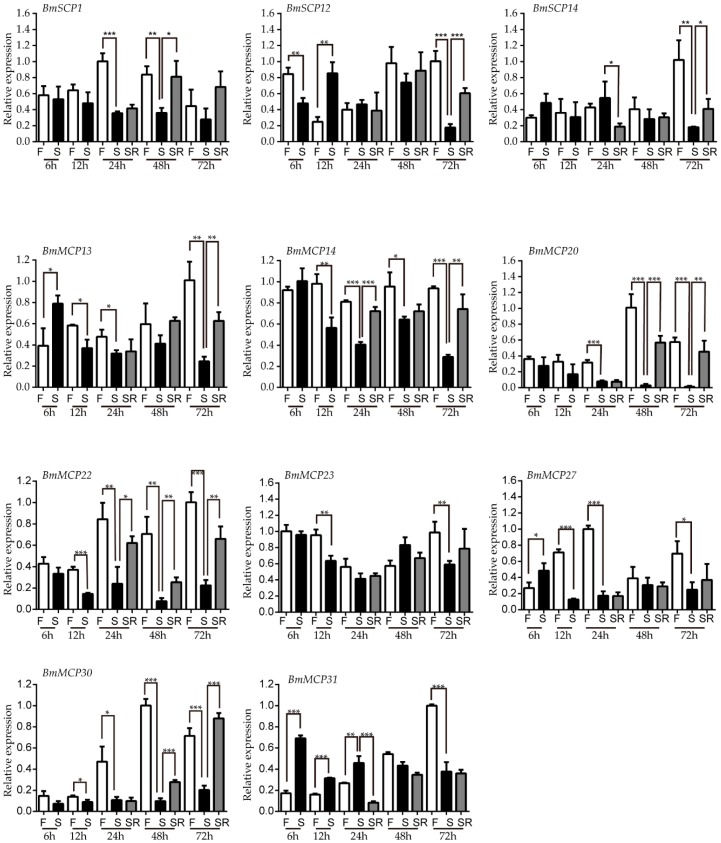
Expression patterns of midgut-specific carboxypeptidases after feeding, starvation and starvation-refeeding. Differences among groups were identified by Student’s *t*-test. * *p* < 0.05; ** *p* < 0.01; *** *p* < 0.001. Abbreviations: F, feeding; S, starvation; SR, starvation-refeeding.

As shown in Figure 4, we tested the influence of feeding, starvation and starvation-refeeding on the expression level of midgut-specific carboxypeptidases (Figure 4). Our results showed that these carboxypeptidases were significantly downregulated after starvation and restored their expression after re-feeding.

The expression of carboxypeptidases in the feeding group was stable throughout the experimental period. The expression level of *BmSCP12*, *BmSCP14*, *BmMCP13*, *BmMCP20*, *BmMCP22*, *BmMCP30* and *BmMCP31* reached a peak between 48 and 72 h after feeding, whereas that of other carboxypeptidases, including *BmSCP1*, *BmMCP23* and *BmMCP27*, reached a peak much earlier. *BmMCP14* was highly expressed throughout the starvation and refeeding period, whereas the expression level of *BmSCP12*, *BmMCP13*, *BmMCP27* and *BmMCP31* was significantly increased at the beginning of starvation. This may be the stress response of silkworm midgut. Eleven carboxypeptidases were significantly downregulated after starvation; most of them (*BmSCP12*, *BmSCP14*, *BmMCP13*, *BmMCP14*, *BmMCP20*, *BmMCP22* and *BmMCP30*) restored their expression level after refeeding, whereas the rest (*BmSCP1*, *BmMCP23*, *BmMCP27* and *BmMCP31*) failed to reach the initial expression level.

## 3. Discussion

Carboxypeptidases are widely found in members of the taxon Metazoa [23,24]. These enzymes are exopeptidases that generally catalyze different reactions based on their active sites. In the present study, 48 carboxypeptidases were identified in the silkworm genome. The silkworm possesses a higher number of carboxypeptidases than do other insects [7,25]; therefore, further study is needed to investigate their unknown functions.

The size of silkworm carboxypeptidases ranges from 73 AA–1051 AA. It is generally considered that 40–50 residues are the lower limit of the functional domains and that protein sizes range from 40–50 residues to thousands of residues. We predicted the *BmMCP33* (996 AA) and *BmSCP11* (1051 AA) domains and found that the former has two Peptidase_M14 domains, whereas the latter is an endomembrane protein 70. Differences in the size of silkworm carboxypeptidases and the combination of the carboxypeptidase domain with other domains suggested that different carboxypeptidases might have different functions.

According to the domain features, 34 metal carboxypeptidases were classified into two groups: Peptidase_M2 carboxypeptidases and Peptidase_M14 carboxypeptidases [3]. Carboxypeptidases that contain the Peptidase_M2 domain are known as angiotensin-converting enzymes (ACEs) [26,27]. ACEs are highly important for the regulation of blood pressure [28]. *A. gambiae* has nine ACE-like genes, but their functions remain unclear [27]. The M14 family is one of the most widely-studied metal carboxypeptidase subunits. The functions of Peptidase_M14 carboxypeptidases are various and diverse, including the digestion of food [29], the processing of bioactive peptides [30] and the metabolism of bacterial cell walls [31].

Fourteen serine carboxypeptidases were classified into two groups: Peptidase_S10 carboxypeptidases and Peptidase_S28 carboxypeptidases [3]. The Peptidase_S10 carboxypeptidase family is active only at acidic pH and is different from most of the other serine peptidase families [32]. There are two types of Peptidase_S10 carboxypeptidases; one (e.g., carboxypeptidase C) that shows preference for hydrophobic residues [33,34,35] and another (e.g., carboxypeptidase D) that shows preference for basic amino acids on either side of the scissile bond, but it is also able to cleave peptides with hydrophobic residues [33,36,37]. Carboxypeptidases in the family S28 suppress angiotensin II by the cleavage of the C-terminal-Pro Phe bond [38]. Additionally, recombinant carboxypeptidases in the family S28 associated with H-kininogen are able to activate plasma prekallikrein [39]. In general, serine carboxypeptidases are considered to play the role of lysosomes and participate in the turnover of proteins. In addition, some of them release amino acids from extracellular proteins and peptides [3].

Phylogenetic analysis of silkworm carboxypeptidases is presented in Figure 1. The tree of metal carboxypeptidase topologies indicated that the divergence and duplication of the Peptidase_M14 carboxypeptidase gene occurred before the separation of *B. mori* and *A. aegypti*. Eleven *A. aegypti* carboxypeptidase genes were induced in the midgut by blood-meal feeding [7], suggesting that *B. mori* carboxypeptidase genes might be also induced by food intake. The silkworm Peptidase_M14 carboxypeptidase is very conservative in the taxon Metazoa. Human digestive carboxypeptidases (NP_001859) hydrolyze the C-terminal peptide of dietary polypeptide chains [40]. Therefore, the silkworm Peptidase_M14 carboxypeptidase might also have a digestive function. Similarly, the *B. mori* Peptidase_S28 carboxypeptidase genes and the *T. brasiliensis* serine carboxypeptidase genes share orthologs in the main branch of the tree. *T. brasiliensis* use serine carboxypeptidase as a digestive enzyme, suggesting that the *B. mori* Peptidase_S28 carboxypeptidase might be also a digestive enzyme.

Carboxypeptidases play key roles in various physiological and biochemical processes in many insects. In the present study, some Peptidase_M14 carboxypeptidases and the serine carboxypeptidases *BmSCP1*, *BmSCP3*, *BmSCP12* and *BmSCP14* were specifically expressed in the midgut of silkworm. In *Anopheles culicifacies*, the carboxypeptidase AcCP is specifically expressed in the midgut, whereas in *T. brasiliensis*, the serine carboxypeptidases tbscp-1 and tbscp-2 are highly expressed in the posterior midgut (small intestine) and lowly expressed in the salivary glands, fat body and anterior midgut (stomach) [15]. These results demonstrated that silkworm carboxypeptidases might participate in digestion in the midgut. Several carboxypeptidases were specifically expressed in the testis and might play important roles in the male reproductive development. Whereas others were widely expressed in various tissues and might perform different functions. For example, the molting fluid carboxypeptidase A (MF-CPA) is identified in the molting fluid of insects at the pupal ecdysis and molting pre-pupal stages. MF-CPA has been proposed to degrade proteins in old epidermal cells and to participate in the recycling of amino acids [18].

The insect digestive tract is divided into three parts: the foregut, midgut and hindgut. The midgut is the most advanced of digestive organs and the most important place for digestion and absorption in insects. The digestion of silkworm larvae includes mechanical digestion and chemical digestion. Under the action of the midgut digestive juice, macromolecules from mulberry, such as carbohydrates, proteins and lipids, are digested into small molecule compounds and absorbed by midgut epithelial cells. Then, compounds are transported to other organs through the hemolymph to provide energy for silkworm growth, development and other life activities.

In the present study, starvation could regulate the expression levels of carboxypeptidases in the larval midgut, and re-feeding could restore them to the initial levels. These results suggested that the expression of midgut carboxypeptidases was induced by food intake. Similar results have been also reported in *A. aegypti*; 11 of *A. aegypti* carboxypeptidases are upregulated in response to blood meal feeding [7]. The expression profile of induced by starvation and re-feeding in our study was the same as the expression profile of a chymotrypsin-like serine protease in *Spodoptera litura* [41]. Here, the expression levels of *BmSCP1* and *BmMCP30* were higher after re-feeding compared to those during normal feeding. *Harmonia axyridis* can completely compensate the body sizes through accelerated growth [42]. In some animals, the compensatory growth is sometimes faster than the normal growth [43,44], and starvation is applied in animal rearing to obtain economic benefits [45].

In summary, 48 members of the silkworm carboxypeptidase family were identified and characterized in the present study. The expression patterns and two phylogenetic trees of carboxypeptidases were analyzed. We further explored the function of carboxypeptidases, especially of those that were specifically expressed in the midgut. Our findings provided a reference for future studies on *Lepidoptera* carboxypeptidases.

## 4. Materials and Methods

### 4.1. Biological Materials

The silkworm strain *Dazao* (p50) was used in this study. The silkworms were reared on fresh mulberry leaves at 25 °C with 70%–80% relative humidity and a 16-h light/8-h dark cycle in a growth chamber of the State Key Laboratory of Silkworm Genome Biology. Samples from embryonic stages and larval tissues were isolated and stored in liquid nitrogen.

### 4.2. Identification of the Carboxypeptidase Family in Silkworm

SilkDB [46] was used to predict the silkworm carboxypeptidase family. Carboxypeptidase genes from other species were downloaded from GenBank [47]. The BLAST alignment tool was downloaded from the ftp site of the National Center for Biotechnology Information [48]. Carboxypeptidases sequences from other species were used as queries to BLAST against the SilkDB with an E-value threshold of 10^−6^ [49]. Subsequently, SMART (Simple Modular Architecture Research Tool) [50] and Pfam [51] were used to validate each putative protein.

### 4.3. Bioinformatics and Phylogeny Analysis of the Silkworm Carboxypeptidase Family

The open reading frame (ORF) of carboxypeptidases in *B. mori* was identified using ORF Finder [52]. The signal peptide was predicted by SignaIP 4.1 [53]. The molecular weight and isoelectric point were predicted using ProtParam [54]. The amino acid sequences of putative carboxypeptidase were aligned using ClustalX [55]. A phylogenetic trees of metal-carboxypeptidases and another of serine-type carboxypeptidases were constructed by the neighbor-joining method with 1000 bootstrap replicates using MEGA 6.0 [56,57].

### 4.4. Expression Profiles of Silkworm Carboxypeptidase Genes via Whole-Genome Microarrays

A genome-wide oligonucleotide microarray with more than 22,000 probes, including 44 carboxypeptidase-specific oligonucleotide probes, was established as previously described [58]. We identified four carboxypeptidase genes without specific oligonucleotide probes in the database. Microarray data revealed that carboxypeptidase genes had different expression patterns in the tissues of the fifth instar larva at Day 3. Next, diverse tissues, including testis, ovary, head, integument, fat body, midgut, hemocytes, Malpighian tubules, anterior/middle silk gland and posterior silk gland, were collected. To identify the developmental expression patterns, silkworm from 20 different time points (from Day 3 of the fifth instar larval stage to the moth stage) were collected from both genders. Gene expression levels were visualized using GeneCluster 3.0 (University of Tokyo, Tokyo, Japan) [59].

### 4.5. Silkworm Starvation Experiment

Newly molted fifth instar larvae were divided into three groups, to test whether carboxypeptidases were induced by starvation. Larvae in the feeding group were fed on mulberry leaves throughout the experimental period. Larvae in the starvation groups were starved for 6, 12, 24, 48 and 72 h. Larvae in the starvation-refeeding groups were starved for 12, 24 and 36 h and then fed for 12, 24 and 36 h, respectively [41,60]. The larval midguts in each group were collected for analysis.

### 4.6. RNA Extraction

Total RNA was extracted from all tissues (testis, ovary, head, integument, fat body, midgut, hemocytes, Malpighian tubules, anterior/middle silk gland and posterior silk gland) at Day 3 of the fifth instar larval stage and starvation experiment samples using the Total RNA Kit II (Omega, Norcross, GA, USA), according to the manufacturer’s protocol. Total RNA (2 μg) was reverse transcribed into cDNA using M-MLV reverse transcriptase (Promega, Madison, WI, USA). To synthesize the first-strand cDNA, 2 μg of total RNA was mixed with 2 μL of 50 μM oligo (dT) in a total volume of 15 μL. The mixture was briefly spun, heated at 70 °C for 5 min and incubated on ice for 5 min. The mixture was then spun briefly and replaced on ice. After other components (5 μL 5× first strand synthesis buffer, 1 μL dNTP mix, 1 μL RNase inhibitor, 100 U M-MLV reverse transcriptase) were added to the mixture, the reaction mixture was spun briefly and incubated at 42 °C for 1.5 h. The cDNA was then incubated at 92 °C for 10 min and stored at −20 °C.

### 4.7. qRT-PCR

qRT-PCR was performed using the Step-One-Plus™ Real-Time PCR system (Thermo-Fischer Scientific, Waltham, MA, USA) with SYBR^®^ Premix Ex Taq™ II (TaKaRa, Shiga, Japan). PCR conditions were 94 °C for 30 s, followed by 40 cycles at 95 °C for 5 s and 60 °C for 30 s. PCR conditions were 94 °C for 30 s, followed by 40 cycles at 95 °C for 5 s and 60 °C for 30 s. All cDNA samples were normalized using the *B. mori* eukaryotic translation initiation factor 4A (BmeIF-4a, silkworm microarray probe ID, sw22934; sense primer, 5′-TTCGTACTGGCTCTTCTCGT-3′; antisense primer, 5′-CAAAGTTGATAGCAATTCCCT-3′) as the internal control. Each expression assay was repeated at least three times. The primer sequences of all genes are listed in Table S1. The relative gene expression level was determined by the 2^−ΔΔ*C*t^ method [61]. Statistical significance at *p* < 0.05 was determined by Student’s *t*-test using GraphPad [62].

## Figures and Tables

**Table 1:** Characterization of silkworm carboxypeptidases.

Gene	Name in SilkDB	Chromosome	Scaffold	Position	Strand	Domain	Size (AA)	Probe	*M*_W_/Da	pI	Signal Peptide
*BmMCP1*	BGIBMGA002530-TA	9	nscaf2511	5048133–5052368	+	Peptidase_M2	73	sw03136	8984.4	9.3	no
*BmMCP2*	BGIBMGA006234-TA	6	nscaf2851	1576285–1581353	−	Peptidase_M2	409	sw03231	46,754.2	4.89	no
*BmMCP3*	BGIBMGA002527-TA	9	nscaf2511	4990965–5002524	+	Peptidase_M2	649	sw14172	74,229.8	5.11	yes
*BmMCP4*	BGIBMGA006539-TA	6	nscaf2853	6801913–6813068	+	Peptidase_M2	647	sw11710	74,185.1	5.39	no
*BmMCP5*	BGIBMGA002529-TA	9	nscaf2511	5030694–5035485	+	Peptidase_M2	173	sw20193	–	–	no
*BmMCP6*	BGIBMGA002531-TA	9	nscaf2511	5192136–5202359	+	Peptidase_M2	153	sw11006	17,724	7.01	no
*BmMCP7*	BGIBMGA003228-TA	2	nscaf2623	1029259–1049209	−	Peptidase_M2	711	sw17925	82,533.6	9.06	no
*BmMCP8*	BGIBMGA002526-TA	9	nscaf2511	4965783–4980141	+	Peptidase_M2	648	sw00643	74,913.1	5.34	yes
*BmMCP9*	BGIBMGA009693-TA	2	nscaf2964	820517–835616	−	Peptidase_M2	535	sw19815	62,129.8	7.32	no
*BmMCP10*	BGIBMGA009487-TA	14	nscaf2953	517470–526524	+	Peptidase_M14	428	–	48,175.4	6.06	yes
*BmMCP11*	BGIBMGA004799-TA	25	nscaf2818	2043455–2064130	−	Peptidase_M14	468	sw03973	53,767.3	9.18	no
*BmMCP12*	BGIBMGA004800-TA	25	nscaf2818	2024210–2029825	−	Peptidase_M14	344	sw20598	38,598.6	6.55	no
*BmMCP13*	BGIBMGA013275-TA	16	nscaf3063	3241878–3245274	+	Peptidase_M14	346	sw18892	39,898	5.24	yes
*BmMCP14*	BGIBMGA013276-TA	16	nscaf3063	3256098–3269527	+	Peptidase_M14	445	sw05014	50,709.9	5.41	yes
*BmMCP15*	BGIBMGA004801-TA	25	nscaf2818	1996486–2007642	−	Peptidase_M14	427	sw18974	48,293.5	5.78	no
*BmMCP16*	BGIBMGA008973-TA	3	nscaf2930	2150368–2156343	+	Peptidase_M14	365	–	41,363.1	9.22	no
*BmMCP17*	BGIBMGA001892-TA	19	nscaf2204	1727896–1729970	−	Peptidase_M14	298	sw09352	33,757.5	6.37	no
*BmMCP18*	BGIBMGA001891-TA	19	nscaf2204	1730646–1734010	−	Peptidase_M14	418	sw14852	48,425.2	9.24	no
*BmMCP19*	BGIBMGA008975-TA	3	nscaf2930	2171627–2175156	+	Peptidase_M14	267	sw07649	30,547.4	5.31	no
*BmMCP20*	BGIBMGA009477-TA	14	nscaf2953	493144–498033	−	Peptidase_M14	429	sw02069	48,772.3	6.08	yes
*BmMCP21*	BGIBMGA004797-TA	25	nscaf2818	2109792–2113829	−	Peptidase_M14	383	sw11839	43,402.5	5.51	no
*BmMCP22*	BGIBMGA009478-TA	14	nscaf2953	480242–485505	−	Peptidase_M14	394	sw00546	44,578.6	5.39	no
*BmMCP23*	BGIBMGA004798-TA	25	nscaf2818	2099617–2105069	−	Peptidase_M14	395	sw03321	44,319.1	4.8	no
*BmMCP24*	BGIBMGA006871-TA	10	nscaf2859	1283815–1295739	+	Peptidase_M14	604	sw12842	68,595.4	6.45	yes
*BmMCP25*	BGIBMGA008976-TA	3	nscaf2930	2176287–2190645	+	Peptidase_M14	365	sw03831	41,702.1	6.15	no
*BmMCP26*	BGIBMGA008910-TA	3	nscaf2930	1816975–1823616	−	Peptidase_M14	479	sw07559	53,979.4	5.75	yes
*BmMCP27*	BGIBMGA004830-TA	25	nscaf2818	155397–165470	−	Peptidase_M14	670	sw03365	75,788.8	8.55	no
*BmMCP28*	BGIBMGA006715-TA	10	nscaf2855	3350086–3361536	+	Peptidase_M14	371	sw20772	42,903	5.3	no
*BmMCP29*	BGIBMGA001890-TA	19	nscaf2204	1746161–1749490	−	Peptidase_M14	353	sw20818	40,328.8	6.09	no
*BmMCP30*	BGIBMGA009486-TA	14	nscaf2953	512395–515181	+	Peptidase_M14	409	sw10048	46,594.4	5.81	no
*BmMCP31*	BGIBMGA009476-TA	14	nscaf2953	546619–551421	−	Peptidase_M14	397	sw12317	45,444.3	6.06	no
*BmMCP32*	BGIBMGA000307-TA	22	nscaf1681	1661872–1673460	−	Peptidase_M14	483	sw11693	54,330.3	5.73	yes
*BmMCP33*	BGIBMGA012807-TA	16	nscaf3058	6657227–6684446	−	Peptidase_M14	996	sw17444	11,1592	5.73	no
*BmMCP34*	BGIBMGA012806-TA	16	nscaf3058	6684947–6690407	−	Peptidase_M14	294	–	33,318.1	5.55	yes
*BmSCP1*	BGIBMGA012452-TA	9	nscaf3045	2246700–2252046	−	Peptidase_S28	352	sw12542	40,622	4.69	yes
*BmSCP2*	BGIBMGA003579-TA	5	nscaf2674	1284051–1285310	−	Peptidase_S28	419	sw13803	48,709.9	4.96	no
*BmSCP3*	BGIBMGA008167-TA	24	nscaf2891	256973–274367	+	Peptidase_S28	282	sw14786	31,433.3	4.83	no
*BmSCP4*	BGIBMGA000549-TA	1	nscaf1690	3748863–3757879	−	Peptidase_S28	439	sw20204	49,930.5	6.08	yes
*BmSCP5*	BGIBMGA013534-TA	5	nscaf3075	927568–931683	+	Peptidase_S28	383	sw14860	42,863.6	6	no
*BmSCP6*	BGIBMGA003110-TA	4	nscaf2589	2567114–2581357	+	Peptidase_S10	374	sw00555	43,079.3	6.05	no
*BmSCP7*	BGIBMGA012773-TA	16	nscaf3058	8186538–8187953	−	Peptidase_S10	471	sw04192	53,120.8	5.56	yes
*BmSCP8*	BGIBMGA003111-TA	4	nscaf2589	2593763–2602101	+	Peptidase_S10	402	sw17614	45,191.6	5.11	no
*BmSCP9*	BGIBMGA003109-TA	4	nscaf2589	2546587–2560291	+	Peptidase_S10	285	–	32,834.9	5.7	no
*BmSCP10*	BGIBMGA013085-TA	16	nscaf3058	8193345–8194772	+	Peptidase_S10	475	sw14878	54,378	8.46	yes
*BmSCP11*	BGIBMGA003112-TA	4	nscaf2589	2615158–2633253	+	Peptidase_S10	1051	sw01972	11,9508	5.66	no
*BmSCP12*	BGIBMGA010348-TA	12	nscaf2990	809983–812392	+	Peptidase_S10	403	sw06212	46,108.2	4.99	no
*BmSCP13*	BGIBMGA006502-TA	6	nscaf2853	4266715–4273371	+	Peptidase_S10	376	sw15928	42,942.2	8.8	no
*BmSCP14*	BGIBMGA010349-TA	12	nscaf2990	814696–818250	+	Peptidase_S10	365	sw06213	40,585.3	4.95	no

## Supplementary Materials

Supplementary materials can be found at http://www.mdpi.com/1422-0067/17/8/1203/s1.
