# Supplementary material for: Genome-Wide Identification and Characterization of Carboxypeptidase Genes in Silkworm (Bombyx mori)
Source: Int J Mol Sci. 2016 Jul 28;17(8):1203. doi: 10.3390/ijms17081203 (PMC5000601; doi:10.3390/ijms17081203)
Supplement: Supplementary file 1 [file ijms-17-01203-s001.pdf]

Supplementary Materials: Genome-Wide Identification and Characterization of Carboxypeptidase Genes in Silkworm (*Bombyx mori*)

Junhong Ye, Yi Li, Hua-Wei Liu, Jifu Li, Zhaoming Dong, Qingyou Xia and Ping Zhao

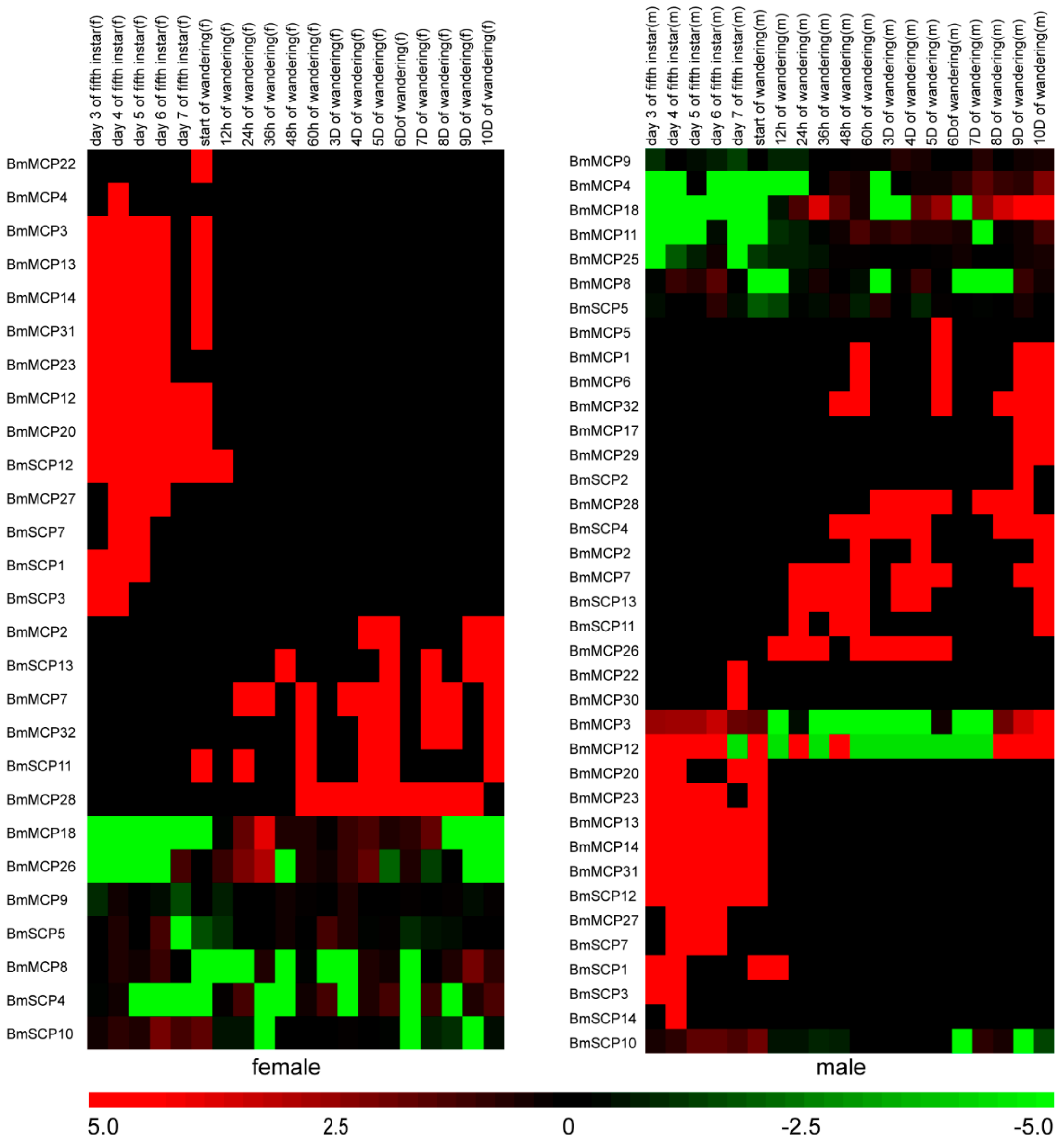

Figure S1. Microarray analysis of silkworm carboxypeptidases at different developmental stages. Each column represents 20 different time points: on days 3, 4, 5, 6, and 7 of the fifth instar stage, start of wandering, 12, 24, 36, 48, 60, 72, 96 and 120 h after wandering; days 6, 7, 8, 9, and 10 after wandering, and adult. Red represents high expression, and green represents lower expression.

Table S1. Primer sequences for quantitative real-time PCR.

Gene	Primers	Sequence (5′to 3′)
BmMCP12	Forward	TTCTCGGAAGTGGAAACAAG
	Reverse	AGTGCTGTGAGCGAAAGGAA
BmMCP13	Forward	CTGGCGAAATGGAGGAGAAA
	Reverse	TAGGTTGACGGAACGGGTGC
BmMCP14	Forward	GCCCAGGATTACGGTCAGCA
	Reverse	CGATTCCCTTCCAGGTCTCC
BmMCP20	Forward	CAACCCTGATGGATACGAAC
	Reverse	CAACGCATTAGGAGGAAGCT
BmMCP22	Forward	CAGGGAGGCAAGGGCAGATT
	Reverse	ACGGTGACGGTTTCAGGGTA
BmMCP23	Forward	CAAACTTATGCTGGACCTTC
	Reverse	CTGGGAATAGGAGTGGAATG
BmMCP27	Forward	ACAGACGACTAACCGAGGGC
	Reverse	TCAAAGATGATGGAGCAAGG
BmMCP30	Forward	TATTCGGAAGGGACGGACTG
	Reverse	TAAATCGGGATAGCGGTGAG
BmMCP31	Forward	GACTCTAGCTCCAGTCCCTG
	Reverse	GCTGCCTAAACATCCCACTA
BmSCP1	Forward	CCTTGTGGGAGGTGAATGGA
	Reverse	GTTTGCCCGTAATACCGATG
BmSCP3	Forward	AGTGGCTTTGGTTGGGTGTT
	Reverse	TTTGTCCGCCTTGTACCTTG
BmSCP12	Forward	GGGGCTCACAGTCTGGTAAT
	Reverse	TTCTGGGTTGTCCCTTGGTA
BmSCP14	Forward	AGGTGAAGGCATTGGTGATA
	Reverse	GCCAGTAGACTAGGCGGTAC
sw22934	Forward	TTCGTACTGGCTCTTCTCGT
	Reverse	CAAAGTTGATAGCAATTCCCT
